# Emerging, hybrid & smart composites

**DOI:** 10.1186/s42252-021-00028-y

**Published:** 2021-12-31

**Authors:** Christophe Binetruy, Véronique Michaud

**Affiliations:** 1 École Centrale de Nantes, Research Institute in Civil Engineering and Mechanics (GeM), UMR CNRS 6183, 44321 Nantes Cedex 3, France; 2 Head of Laboratory for Processing of Advanced Composites (LPAC), MXH145, Station 12, Ecole Polytechnique Fédérale de Lausanne (EPFL), 1015 Lausanne, CH Switzerland

Functional composite materials in principle advance the field of composites beyond their specific structural properties. The intrinsically heterogeneous nature of composite materials allows additional material phases to be introduced, with specific added properties such as electrical conductivity, magnetic properties, or integrated surface functions. While significant progress has been made on these hybrid material systems, a complete understanding of their properties is necessary to tailor their functions for a given application. Specific challenges and opportunities fuel this dynamic research area.

This special collection in *Functional Composite Materials* highlights the diversity in contributions, class of materials, emerging areas, cross-disciplinary applications, specific challenges, and opportunities in this emerging. The collection features a subset of papers originally selected to be presented at the 19th European Conference on Composite Materials (ECCM19) in June 2020 in Nantes, France, which was cancelled as a result of the COVID-19 pandemic.

The field of functional composites is very broad as it consists in bringing several functions to composites to satisfy complex specifications. This great diversity is illustrated in this collection, where various technologies and constitutive materials are employed to bring the desired properties to composites. As a result, this collection by no means pretends to fully cover the field, but instead to bring to light some interesting materials related aspects in this vibrant field.

One approach to reach functional composite materials consists of hybridizing materials featuring complementary functional properties. In [1], the in-plane and transverse electrical conductivities of Carbon-Fiber-PEEK composites are significantly improved by nanomaterial functionalization via spray deposition of exfoliated graphene nanosuspensions. Adding only a few wt% graphene in the interlayers of structural CF-PEEK composites by this technique led to more than 60% increase in transverse electrical properties, and much more in plane.

In [2], a more macroscopic approach was followed to improve the longitudinal electrical conductivity of structural composites, by braiding carbon and metallic fibers, while the through-thickness conductivity was achieved by tufting conductive fibers into the braided fibrous structure, bringing both functional and structural benefits for through thickness properties. This approach was modelled by Finite Element techniques to provide a guide to evaluate the functional performance of the braided hybrid materials.

The tufting technology was also used in [3] to improve the through-thickness mechanical strength of flax fibre reinforced green bio composites, using glass fibers. This study confirms that the mechanical functionalization of a laminated structure results in the search for a compromise between improvement in the thickness direction and the reduction in performance in the planar direction.

Combining dissimilar materials sometimes also involves the addition of an interlayer to ensure chemical compatibility in novel welding techniques. This point is illustrated in [4] for the fusion welding of thermoset to thermoplastic polymer composites. The thermoset side needs to be functionalised through co-curing with a thin thermoplastic interlayer, and so several grades of PEEK and PEI were evaluated, in addition with a prior plasma treatment. The main remaining challenge is the fine tuning of the Infra-red welding parameters, to avoid degradation in final properties as compared to more conventional welding techniques.

The functionalization of materials allows the development of sensors adapted to numerous applications. The structural health of polymer composites was monitored in [5] by inserting carbon fibre rovings into glass non-crimp fabrics. It was shown that the capacitance decrease is highly correlated to the crack formation, as the permittivity of air gaps is lower than that of the composite. This method is promising for crack detection in structures, as it does not compromise the structural properties of the glass laminates, and it can be implemented in critical locations.

The development of more reliable magnetic sensors was investigated in [6]. Permanent magnets are usually bonded to metal substrates, which limits their lifetime and operating temperature. The authors show that it is possible to develop more reliable sensors by torsional ultrasonic welding of the magnets to stainless steel substrates.

In parallel, there is a growing interest in the use of natural materials in composite structures, in response to environmental issues. In [7], the mechanical and hygroscopic properties of thermoformed Chemi-Thermo-Mechanical Pulp were analysed as a function of manufacturing parameters. These functional properties are key for packaging applications. Results showed that a careful optimisation of the process parameters led to a lower water uptake of the material, with better retention of mechanical properties even after moisture exposure. Scholz et al [8] investigated application-oriented adaptive elements based on cellulose Cottonid capable of complex, passive, moisture-induced shape changes with adjustable amplitude. For construction-related applications, solar radiation was studied as an environmental trigger on this cellulose-based humidity-sensing material. In conclusion, promising tailor made natural material sensors can be produced.

Finally, in [9], the influence of the temperature and humidity of various seasons on the curing cold mix asphalts was studied. Cold mix asphalts are better in terms of environmental impact as they do not require high temperature to be processed and produce less fumes. However, their mechanical performance over time is still not fully understood, and results of the presented study showed that warm and dry temperatures tend to increase the material stiffness as well as the ageing of the binder material.
